# New frontiers in regenerative medicine: Protocol standardization and morphological assessment of leukocyte-platelet rich fibrin in cows, with a longitudinal study on growth factor release dynamics

**DOI:** 10.1016/j.vas.2025.100564

**Published:** 2025-12-29

**Authors:** Giovanni Della Valle, Maria Chiara Alterisio, Jacopo Guccione, Chiara Caterino, Federica Aragosa, Gianmarco Ferrara, Davide De Biase, Paolo Ciaramella, Gerardo Fatone

**Affiliations:** a Department of Veterinary Medicine and Animal Production, University of Naples Federico II, 80137 Naples, Italy; b Department of Veterinary Sciences, University of Messina, 98100 Messina, Italy; c Department of Pharmacy/DIFARMA, University of Salerno, 84084 Fisciano, Salerno district, Italy

**Keywords:** Leukocyte-platelet-rich fibrin, Bovine, Regenerative medicine, Healing, One-health

## Abstract

Leukocyte-Platelets Rich Fibrin (L-PRF) is a platelet-derived autologous agent. It is a powerful wound-sealing biological scaffold that promotes the healing process through the release of growth factors (GFs) and matrix proteins. To date, the l-PRF in bovines has not yet been investigated and a standardized production protocol has not yet been described. Therefore, the aim of the study was to standardise the l-PRF production protocol and to evaluate macroscopically and histologically the l-PRF membranes obtained and observe the GFs release over time and lactation phases: at 1 h (T0), 4 h (T1), 24 h (T2), and 168 h (T3) after extraction. Eighty cows were divided into four groups of 20 animals, based on their lactation phase. A whole blood sample of 20 ml was taken and divided for l-PRF clot production and haemato-biochemical investigations. The membranes presented the following overall mean characteristics: a length of 34.4 ± 7.3 cm, a width of 12.5 ± 2.6 cm, and a weight of 4.9 ± 0.17 cm. Histological analysis showed a well-defined histoarchitecture consisting of a first layer of erythrocytes, a layer of leukocytes, erythrocytes, and platelets, a layer of leukocytes, a layer of platelets, and finally a layer of slightly eosinophilic fibrin. The GFs significantly increased with upward trends in all phases of lactation and at each time control, overlapping the behavior of GF in other species. Based on the encouraging findings of this study, further clinical trials for applicative potential of the l-PRF membrane in bovine medicine should be assessed.

## Highlights

• This study provides the first in-depth investigation into autologous l-PRF membranes derived from bovine blood.
• This study provides the standardisation of the l-PRF production protocol.
• Our results highlight the membranes’ promising macroscopic, histological, and biological characteristics, alongside a dynamic release of growth factors over time.
• The study opens exciting possibilities for regenerative medicine in dairy cattle, particularly in supporting wound healing and reducing antimicrobial use, aligning with the One Health approach.

## Introduction

Platelet concentrates (PCs) are biological autologous blood products that have been widely used since their discovery and introduction in human dental and maxillofacial surgery(Choukroun, Fabien Adda et al., 2001) (Choukroun, F Adda et al., 2001)**,** as well as in sports and orthopedic medicine (Amable et al., 2013; Miron et al., 2017). Supraphysiological concentrations of platelets and related growth factors (GFs) commonly housed in their α-granules constitute the main component of PCs (Dohan et al., 2006b). According to the classification of Dohan et al., (Dohan et al., 2006a, 2006b), PCs are divided into two main categories based on the fibrin architecture: platelet-rich plasma (PRP) with a low-density fibrin network, and platelet-rich fibrin (PRF) with a high-density fibrin network. Both PRP and PRF were further divided into Pure-PRP (P-PRP) and Leukocyte-PRP (L-PRP), as well as Pure-PRF (P-PRF) and Leukocyte-PRF (L-PRF), depending on the presence or absence of leukocytes (Dohan et al. 2006a). Unlike PRP, which utilises blood with an anticoagulant, PRF is prepared using whole blood. Different methods enable the preparation of PCs, including gravitational centrifugation, standard cell separators, and autologous selective filtration techniques. (Harrison 2018). l-PRF is a 2nd-generation platelet-derivate bioscaffold achieved by a whole physiological process, promoted by centrifugation, and characterized by a relevant concentration of platelets releasing GFs in the tissues surrounding the point of application such as platelet-derived growth factor type AA, AB and BB (PDGF-AA, PDGF-AB, PDGF-BB), transforming growth factor-β1 (TGF-β1), vascular endothelial growth factor (VEGF), as well as other important proteins (e.g., basic fibroblastic grow factor, connective tissue grow factor, etc.) (Miron et al. 2017; De Pascale et al. 2015).

The effect of several PRF formulations on fibroblasts involved in soft tissues wound healing, endothelial cells, and GFs release was mainly investigated in vitro and in vivo in human medicine (Kobayashi et al. 2016). Depending on its application, PRF has been shown to induce the proliferation of dermal and gingival fibroblasts, keratinocytes, and extracellular matrix collagen type 1, enhancing both soft and hard tissue healing (Lundquist et al., 2008, 2013; Clipet et al., 2012; De Pascale et al., 2015; Kobayashi et al., 2016).

In recent years, the use of PCs families has spread in bovine medicine, although the field remains relatively underexplored (Caterino et al. 2023). In this ruminant, PRP's beneficial effects were demonstrated when applied in the treatment of some diseases of the reproductive system, in-vitro production of embryos or in the attempt to reduce the use of antibiotics in cows affected by subclinical mastitis (Carmona & López 2024; Constant et al. 2023; Duque-Madrid et al. 2021; Lange-Consiglio et al. 2014, 2015; López et al. 2024; Ramos-Deus et al. 2020). Additionally, equally positive effects on the healing process of sole ulcers through the combined use of gelatin microspheres incorporated with PRP and alginate were described (Tsuzuki et al. 2012), as well as a favorable impact on articular cartilage and ligament healing (Petrera et al., 2013; Xie et al., 2015; Tambella et al., 2018). Lastly, the protective effects of autologous PRP combined with antibiotics were assessed in vitro on chondrocytes from animals with septic arthritis caused by *Staphylococcus aureus* infection (Muir et al., 2021). If, on the one hand, the amount released from PRP and the comparison of platelets and white blood cell concentrations in its layers and supernatant were described for the bovine (Gutiérrez et al. 2017), on the other hand, the comparison of GFs release over time and at different phases of lactation has not yet been investigated in this species. In human medicine, it has been shown that the PRF releases GFs consistently over time, while the PRP releases a high level of GFs mainly during the first 24 h (Ghanaati et al. 2014; Kobayashi et al. 2016); the same results have been reported in canine medicine (Caterino et al. 2022), but no information is available for cows.

Based on considerations made so far, the PRF has the potential to find widespread application in the buiatric clinical setting, naturally enhancing the healing processes and potentially reducing antibiotic use (Caterino et al. 2023). However, to the best of the authors’ knowledge, a standardised production protocol for l-PRF in the bovine species has not yet been described. Although some protocols to produce l- PRF have been described in other animal species such as horses, dogs and cats (Caterino et al. 2022; Crisci et al. 2017; Soares et al. 2021), it is not possible to apply them, as is, to cattle due to the long-know differences in sedimentation rate of bovine platelets (Clemmons et al. 1983). Therefore, the aims of current study are multiple: (i) to standardize the production of clots and l-PRF membranes; (ii) to performed macroscopic and histological analyses of the membranes obtained; (iii) to evaluate the quality and quantity of the GFs released by in vitro analysis over time and at different lactation phases by means of a subsequent observational study.

## Materials and methods

### General

The present clinical study, designed as a longitudinal observational was carried on from March to June 2023 in the Southern Italy. The study population was made up of 80 pluriparous (≥2 lactations), Holstein Friesian dairy cows housed in a free-stall barn.

A correlation analysis with a 0.90 power level, a two-tailed significant level of 0.05, and an assumed effect size of 0.35 were considered for the samples size calculation, as reported by Friedman (1982) (Friedman 1982). The study received institutional approval from the Ethical Animal Care and Use Committee of the University of Napoli Federico II (PG/2017/0099,607). All the procedures performed during the study abode by the standard *good clinical practices* (*EMA, European Medicine Agency. VICH GL9:*) and were performed by expert clinicians. Finally, the farmer was informed and in agreement with the purposes and methods (following STROBE guidelines - http://www.strobe-statement.org) giving a written consent for the study.

### Farm and management

The selected cows farm was randomly extracted within 6 regularly requesting consultancy services at the Veterinary Teaching Hospital – Didactic Mobile Clinic Service of the Department of Veterinary Medicine and Animal Production – University of Napoli Federico II (Italy) having similar characteristics, Briefly: (i) milking herd consisting of <300 heads; (ii) The minimum welfare standard for dairy cows related to housing and the overall management system was guaranteed (iii) presence of in-farm software defining at least cows' days in milk (DIM), (iv) herd health monitoring program which included regular and recorded clinical examination to obtain information on the cows’ health status in the 12 months preceding the study, (v) animals tested as negative by the governmental office for veterinary public health for brucellosis (*Brucella abortus*) and tuberculosis (*Mycobacterium bovis*).

The selected farm consisted of a stable number of milking cows during the 12 months preceding the study (250≤ heads ≤260) fed with total mix ration twice a day, a herringbone parlor to milk the animals twice a day, a 10-month average of 175 ± 25 (DIM ± standard deviation, SD), an average milk yield/head/year of 27.5 ± 4.4 kg, and an average bulk milk somatic cell count values of 300 × 10^3^ ± 89 × 10^3^ cell/mL (mean of the monthly samplings performed the 12 months preceding the study). Moreover, milking cows were kept in two barns of ∼ 600 m^2^ (∼ 20 *m* × 40 m); one intended for animals in early lactation (≤120 DIM) and the other one for middle and late lactation (>120 DIM up to immediately before drying off). Both were characterized by solid grooved concrete floors in the walking and feeding alleys (automatically cleaned four times/day), and free access to the protected water trough was always guaranteed. Late lactation animals were abruptly dried off when they produced <13 kg of milk/day.

### Animals and clinical procedures

All the animals enrolled were pregnant and divided into 4 subsets of 20 heads each one to assess potential effects due to pregnancy and nutrition along lactation (Kononov et al. 2020; Shamloo, Xu & Heilshorn 2012). The classes were: before peak-group (10< BP-*G* ≤ 45 DIM), early lactation-group (45< EL-*G* ≤ 120 DIM), middle lactation-group (between 120< ML-*G* ≤ 200 DIM) late lactation-group (200< LL-*G* <immediately before dry-off). At recruitment time, cows were selected by convenience sampling employing the following eligibility criteria: (i) to be a milking cow in one the lactation phases defined (ii) to be classified as healthy and without health problems since the 6 months preceding the study according to the historical data (i.e., free from systemic diseases or from such affecting individual organs or lameness). Moreover, 48 h (h) before sampling (∼4–5 h after the beginning of feeding time) the overall good health status was confirmed by a complete veterinary clinical examination (e.g. including temperature, breath and beats per minute, etc.,), body condition scoring, locomotor system and a blood sampling (2 ml) (coccygeal venipuncture) for haemato-biochemical investigations. Briefly, some parameters were evaluated directly in farm immediately after collection (3-hydroxybutyric acid and glucose: FreeStyle Optium, Abbott, Chicago, Illinois, US;- iCa2+ and blood gas analysis: i-STAT, Abbott, Chicago, Illinois, US, EG7+ cartridges) and within 1 h at the University Veterinary Teaching Hospital of the Department of Veterinary Medicine and Animal Productions of Napoli (complete blood cell count—HeCo C–Hematology, Radim Seac, Italy). The clinical diagnostic procedures to exclude the presence of any disease originated from a previous study and were partially modified (Fadul et al. 2022). Details are reported within supplementary file 1.

### Blood sampling for L-PRF extraction

For l-PRF clot production, two aliquots of blood [9 mL (mL) each one] were collected by way of coccygeal venipuncture using a Vacutainer™ plastic blood collection tubes (Becton, Dickinson and Company, 9 mL of Plastic Blood Collection Tubes for Trace Element Testing: Serum Clot Activator; Franklin Lakes, US), Vacutainer™ one-use needle and holder (Becton, Dickinson and Company, one-use needle 21 *G* × 7 inches, Franklin Lakes, US). All blood samples were centrifuged within 120 s of collection according to the protocol suggested by Caterino et al. (Caterino et al. 2022).

### Centrifugation protocol and preparation of L-PRF membranes

The centrifugation protocol consisted of acceleration, centrifugation, and deceleration steps and was performed using a table-top centrifuge with a rotor radius of 100 mm (TD4A-WS, In LoveArts). Details including phases, timing and revolution per minute (rpm) are reported in Table 1. A thermometer (TEMP 7 NTC, XS Instruments, Carpi MO - Italy) with a probe (NT 7 L, XS Instruments, Carpi MO - Italy) measured the temperature inside the centrifuge.

**Table 1:** Centrifugation protocol, including timing and RPM/RCF.

**Centrifugation Protocol**		
**Phases**	**Time**	**RCF (g)**
I	30.0 s	Acceleration
II	9.0 min	815
III	11.0 min	644
IV	10.0 min	1006
V	36.0 s	Deceleration
VI	/	blocking

***s***=seconds; **min**=minutes; **rpm**=revolution per minute; **RCF**= Relative Centrifugal Force or **g** force.

**Fig. 1:**
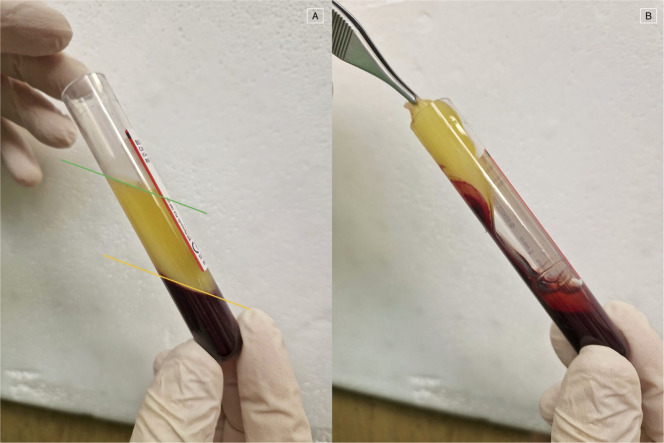
Example of the wound box used for l-PRF membranes extraction (courtesy of Dr. A. Crisci). *A*=top and inferior case; *B*= perforated steel plate; *C*= second-shaped plate.

**Fig. 2:**
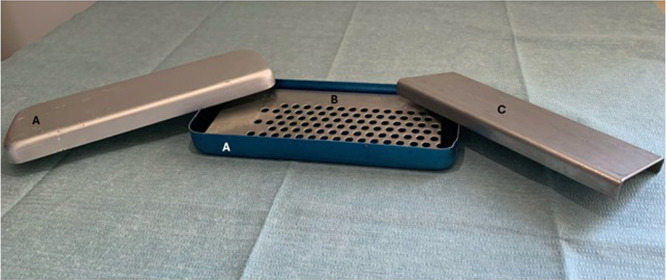
(A) The three fractions of whole blood after centrifugation: platelet-poor plasma (PPP), the liquid layer above the green line; platelet-rich fibrin (PRF), the layer between the green and orange lines; and red blood cells (RBC), below the orange line. (B) Mechanical separation of the RBC fraction from the PRF clot after decantation of the PPP.

Once the centrifugation was completed, in the tube were visible three layers: the platelet-free plasma on the top, the fibrin clot (L-PRF clot) in the middle, and the red clot, consisting of red blood cells, at the bottom. The fibrin clot was gently separated from the red clots by scissors and then placed in the l-PRF Wound Box (Fig. 1, Fig. 2). The wound box is made of a metal container 17.5 × 7.6 × 2 cm containing a perforated steel plate of 150 × 68 × 1.5 mm. A second steel plate acted as a compressor, 150 × 68 × 1.5 mm in size and 148 g in weight. This second-shaped plate exerts a pressure of 142.437 Pa/cm2. Inside the l-PRF Wound Box, the steel plate compressor provided a homogeneous pressure for 15 min.

**Fig. 3:**
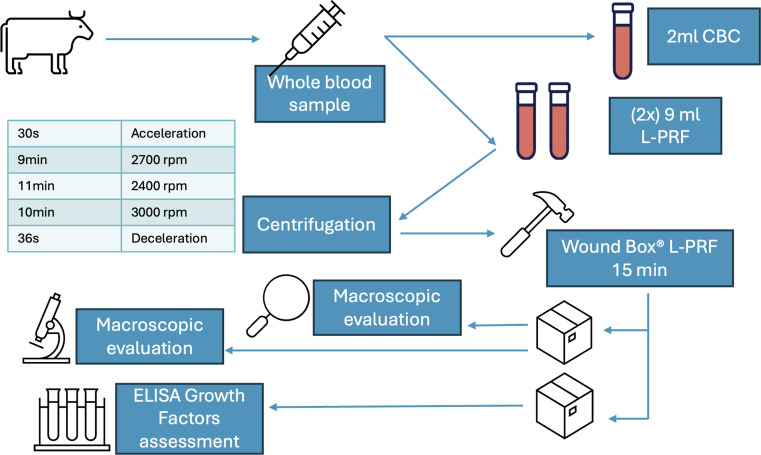
Graphical abstract summarizing the clinical procedure from blood collection to l-PRF membrane production and storage.

All clinical procedures carried out from the blood collection to the membrane production and storage were graphically summarized in Fig. 3.

### Macroscopic and histological investigations

Immediately after formation, the width and length (in centimetres) of each l-PRF membrane were measured using a precision calliper (CDJB15-LTF, Borletti, Italy), and they were weighed (in grammes) using a goldsmith’s scale (PLC200B-C, G&G, Deutschland). The membranes were then stored at room temperature, in two sterile tubes (Demas, tubesin PS, 3 ml, 12 × 55 mm, cod. 00,579,192, Italy), preserved in 10 % neutral buffered formalin (code no 05–01007Q, Bio-Optica, Milan, Italy), for histological analysis, and transported at 4 °C to the laboratory within 6 h for morphological assessment. The Samples were subsequently dehydrated through graded alcohols before being embedded in paraffin wax. For morphological analysis, 5 μm-thick sections were cut and stained with hematoxylin and eosinA semi-quantitative evaluation was also performed for each section by two independent, experienced pathologists in a blind fashion.

The scoring was adapted from Hamed and Hasouni (Hamed & Hasouni n.d.), as follows:

• *Zone layers in the membrane*: the demarcation between membrane layers was described as well (score 1) or poorly demarcated (score 2).
• *Number of cell layers at border***:** the number of cell layers at the periphery of clot was counted as more than ten layers (score 1) or less than ten layers (score 2).
• *Aggregation of cells in the cell layer zone:* the proximity of cells to each other was considered either heavy (score 1) or light (score 2).
• *Cell border morphology:* cell border morphology was evaluated as very clear (score 1), clear (score 2), very unclear (score 3).

#### Growth factors assessment

For GFs assessment by ELISA, 6 membranes originating from BP-G, 5 from both EL-G and ML-G, as well as 6 from LL-G were assessed. These membranes were randomly chosen, taking into account the group’s composition (at least 5 membranes per group). All membranes were placed in a 10 mL tube with 4 mL of sterile Dulbecco’s Modified Eagle’s Medium (DMEM; 11,965,092, Gibco Grand Island, New York 14,072 USA) supplemented with antibiotic/antimycotic (Antibiotic-Antimycotic 100X; 15,240,062, Gibco Grand Island, New York 14,072 USA). Samples were incubated at 37 °C in a humidified 5 % CO_2_ atmosphere. A final centrifugation, performed before the storage, of the 4 mL of DMEM in each tube (15,000 rpm for 10 min) was performed to remove residual particulates. Then, at each time point, the membrane was transferred to a new tube containing 4 mL sterile DMEM, and the previous tube was stored at −20 °C before ELISA quantification. The membrane transfer was done at 1 h (T1), 4 h (T2), 24 h (day 1, T3), 168 h (day 7, T4). Approximately 1 mL of solution was then collected and stored at −20 °C before ELISA quantification.

When all the samples were collected, TGF-β1, insulin-like growth factor-1 (IGF-1), PDGF-AB, and VEGF-A were quantified with commercially available direct Enzyme-Linked-Immunosorbent-Assays (ELISAs) kit (Cat. No. MBS2607521, MyBioSource, Gentaur SRL, Italy). All the assays were validated for the bovine species and were performed according to the manufacturer’s instructions. The results, expressed as optical density (OD), were determined using a spectrophotometer (Multiskan™ FC Microplate Photometer, Thermo Fisher Scientific).

#### Statistical analysis

Throughout the manuscript, data are presented as absolute numbers, percentages, mean ± SD or mean ± standard error of the mean (SEM). Statistical analyses were performed using GraphPad Prism software version 7 (GraphPad Software Inc, La Jolla, CA, USA). Data from macroscopic analysis were evaluated using descriptive statistics, reporting the mean and standard deviation (mean ± SD). All data were tested for normality using the Kolmogorov-Smirnov test. Agreement between the two expert pathologists in the semi-quantitative evaluation of l-PRF membranes was assessed using Cohen’s Kappa Coefficient (κ values between 0.6–0.8 and 0.8–1 indicate good and excellent agreement, respectively) (Altman, 1990). *P*-values < 0.05 were considered statistically significant.

The concentrations of the four growth factors (PDGF-AB, VEGF, TGF-beta, and IGF-1) were treated as dependent variables in a linear mixed model (LMM). IGF-1 data were analyzed on the original scale (ng/mL), as they meet the assumptions of normality and homoscedasticity. In contrast, PDGF-AB, VEGF, and TGF-β1 data were log-transformed (natural logarithm) before LMM analysis to satisfy statistical assumptions.

This approach was used to analyze repeated measures (four blood samples per animal), accounting for the dependency among observations within the same subject. The model included lactation phase (four levels: Early Lactation (EL-G), Late Lactation (LL-G, >120 days), Dry Off (BP-G), and <120 days (ML-G), with early lactation as the reference level) and time of measurement (four levels: T1, T2, T3, T4) as fixed effects, along with their two-way interaction (lactation phase × time). Animal ID was specified as a random effect to model inter-subject variance and the covariance structure. The significance of the fixed effects was evaluated using the F test, with a significance threshold of *p* < 0.05. Post-hoc comparisons of the Estimated Marginal Means (EMMs) were conducted using the Tukey-Kramer HSD test to identify significant differences between factor levels and interaction levels, where appropriate. The Estimated Marginal Means (EMMs) and 95 % confidence intervals (CIs) for the log-transformed factors (PDGF-AB, VEGF, and TGF-β1) were exponentiated (back-transformed using *e*^χ^) and are reported in ng/mL to ensure consistency and biological interpretability of all data in Table 3. The analysis was conducted using JMP statistical software version 18.0.

## Results

### General

All cows with an average lactation number of 2.9 ± 0.9 were included during springtime to ensure good homogeneity of the sampling population.

The haemato-biochemical investigations performed on the 80 cows at enrollment revealed values within the range of normality for the species and phase of production; therefore, they confirmed a good health status of the animals (data are not shown). However, the overall mean value of platelets was 281.7 ± 105.0 (K/μL ± SD), while mean values for the different subsets were 308.9 ± 118.2 for BP-G, 262.7 ± 100.6 for EL-G, 292.5 ± 114.7 for ML-G, and 262.8 ± 83.1 for LL-G. No statistical intergroup differences were detected regarding platelet value. From 80 cows enrolled, a total of 160 blood samples (100 %) were instead collected for l-PRF extraction, which made it possible to obtain as many as 160 clots and related 160 membranes.

### Macroscopic and histological features

**Fig. 4:**
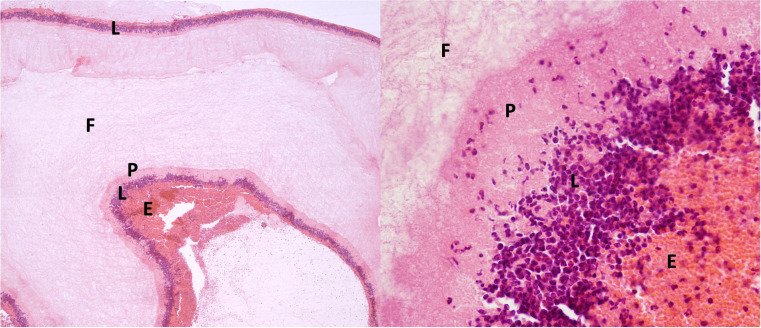
Histology of l-PRF clot. (A) Cellular portion of a l-PRF clot (x4 magnification). (B) =*L*-PRF clot (x40 magnification). *P*=platelets; *E*=erythrocytes; *F*=fibrin, *L*=leukocytes.

Membranes’ sizes and weights are given in Table 2. No statistical differences in size and weight were found between the groups. The Choen's K test showed excellent agreement between the observers (κ = 0.913, *P* < 0.001). Histology analysis allowed us to observe, in all the examined PRF clots, 5 zones or layers according to the distribution and density of blood cells in this biomaterial. The first layer was primarily composed of erythrocytes, followed by a transitional zone of leukocytes and platelets, a layer of fibrin and then a layer of leukocytes and platelets. Erythrocytes were stained red, whereas platelets and aggregates of platelets were stained dark pink. The fibrin network was clearly distinguished by the near absence of color and the scarce presence of cells (Fig. 4).

**Table 2:** Average values of the macroscopic characteristics of the l-PRF membrane extracted using the specific protocol for ruminants. Overall values, as well as values divided by the four subsets, are reported.

	*Macroscopic characteristics*		
	Length *(mm ±SD)*	Width *(mm ±SD)*	Weight *(mm ±SD)*
*All cows*	37.4 ± 7.9	12.5 ± 4.6	4.9 ± 1.6
*BP-G*	35.1 ± 5.3	12.6 ± 3.3	11.8 ± 3.6
*EL-G*	41.0 ± 8.7	12.5 ± 3.6	6.6 ± 2.4
*ML-G*	39.2 ± 10.5	13.2 ± 10.5	0.77±0.5
*LL-G*	34.4 ± 7.3	11.8 ± 1.1	0.59 ± 0.2

BP-*G*=before peak group, EL-*G*=early lactation group; ML-*G*=mid-lactation group; LL-*G*=late lactation group.

### Growth factors

#### Linear mixed model (LMM)

The results of the F tests for the fixed effects and the random effects are summarized in Table 3. Estimated Marginal Means (EMMs) and 95 % Confidence Intervals for all four factors are presented in Table 3. EMMs for PDGF-AB, TGF-β1, and VEGF are reported as exponentiated values (ng/mL) derived from log-transformed data, while IGF-1 EMMs are on the original scale (ng/mL).

**Table 3:** Estimated Marginal Means of Serum Growth Factors by lactation stage and time, derived from the linear mixed model.

Growth Factor	Lactation Stage	Time Point (T)	EMM (ng/mL) (± 95 % CI)
PDGF-AB	Early Lactation	T1 (0 h)	4566.67 (3855.70−5404.10)
(Log-Transformed)	Early Lactation	T2 (2 h)	4813.59 (4068.70−5694.60)
	Early Lactation	T3 (24 h)	5188.76 (4385.60−6134.40)
	Early Lactation	T4 (48 h)	5070.36 (4286.90−5998.60)
	Late Lactation (>120 DIM)	T1 (0 h)	4700.75 (3972.50−5565.80)
	Late Lactation (>120 DIM)	T2 (2 h)	4394.02 (3715.10−5201.70)
	Late Lactation (>120 DIM)	T3 (24 h)	3866.52 (3268.40−4577.10)
	Late Lactation (>120 DIM)	T4 (48 h)	3748.12 (3167.30−4434.70)
	Dry Off	T1 (0 h)	4967.63 (4195.90−5880.80)
	Dry Off	T2 (2 h)	5214.55 (4407.90−6171.70)
	Dry Off	T3 (24 h)	5051.10 (4270.50−5975.30)
	Dry Off	T4 (48 h)	5362.49 (4531.00−6346.30)
—	—	—	:–:
TGF-β1	Early Lactation	T1 (0 h)	0.12 (0.09−0.16)
(Log-Transformed)	Early Lactation	T2 (2 h)	0.17 (0.13−0.22)
	Early Lactation	T3 (24 h)	0.11 (0.08−0.14)
	Early Lactation	T4 (48 h)	0.13 (0.10−0.17)
	Late Lactation (>120 DIM)	T1 (0 h)	0.13 (0.10−0.17)
	Late Lactation (>120 DIM)	T2 (2 h)	0.18 (0.14−0.23)
	Late Lactation (>120 DIM)	T3 (24 h)	0.12 (0.09−0.16)
	Late Lactation (>120 DIM)	T4 (48 h)	0.14 (0.11−0.18)
	Dry Off	T1 (0 h)	0.14 (0.11−0.18)
	Dry Off	T2 (2 h)	0.19 (0.15−0.24)
	Dry Off	T3 (24 h)	0.13 (0.10−0.17)
	Dry Off	T4 (48 h)	0.15 (0.11−0.19)
—	—	—	:–:
VEGF	All Stages	T1 (0 h)	9.96 (7.80−12.60)
(Log-Transformed)	All Stages	T2 (2 h)	10.15 (7.90−12.90)
	All Stages	T3 (24 h)	11.01 (8.60−14.00)
	All Stages	T4 (48 h)	10.10 (7.90−12.80)
—	—	—	:–:
IGF-1	All Stages	T1 (0 h)	2134.68 (1963.80−2305.50)
(Original Values)	All Stages	T2 (2 h)	2174.59 (2003.70−2345.40)
	All Stages	T3 (24 h)	2268.09 (2097.20−2439.00)
	All Stages	T4 (48 h)	2200.77 (2029.90−2371.70)

Values are presented as EMM (ng/mL) with the 95 % confidence interval (CI) in parentheses. The time points (T1, T2, T3, T4) correspond to measurements taken at 0, 2, 24, and 48 h, respectively, after stimulation with leukocyte-platelet rich fibrin (L-PRF). For PDGF-AB and TGF-beta 1, EMMs are provided for each lactation stage × time combination, as the interaction between these factors was statistically significant (*P* < 0.05). For these GFs, EMMs were back-transformed (exponentiated) from log-transformed data to report values in the original unit (ng/mL). For IGF-1 and VEGF, the lactation stage × time interaction was not statistically significant (*P* > 0.05); therefore, EMMs are presented aggregated across all lactation stages ("All Stages") to show the main effect of time only.

The random effect was significant only for TGF-β (*p* = 0.0436), confirming the necessity of the LMM approach for that factor.

Statistical analysis of PDGF-AB) showed strong main effects for lactation phase (*p* = 0.0008) and time (*p* = 0.0080), as well as a significant interaction between lactation phase and time (*p* = 0.0487). This interaction indicates that the kinetic profile of PDGF-AB is not uniform but depends on the specific physiological period of the animal. Post hoc comparisons of the LS Means, performed using Tukey's test, revealed that the kinetic profile of PDGF-AB showed its most significant increase in the early lactation group, with a peak at T3 that differed markedly from the baseline level T1 (*p* < 0.001). Similarly, bovines in the Dry Off phase showed a significant increase in release as early as T2 compared to T1 (*p* < 0.01), suggesting rapid degranulation of platelets in bovines in the non-productive stage. Conversely, in bovines in the late lactation phase (Phase >120 days), a marked reduction in PDGF-AB concentration was observed, which, at the final time point (T4), was significantly lower than the T1 baseline level (*p* = 0.01). This profile suggests a rapid depletion or high utilization rate of the factor in bovines in the advanced stages of production.

The statistical analysis for TGF-β showed strong significance in both main effects (Lactation Phase: *p* = 0.0044; Time: *p* = 0.0002), while the Lactation Phase × Time interaction was not significant (*p* = 0.2792). Post hoc comparisons using Tukey's test on the overall mean concentration of TGF-β revealed that bovines in the Dry Off phase exhibited significantly higher levels compared to those in the early lactation period (*p* < 0.05). This finding indicates that the period of mammary rest is associated with a greater basal concentration or bioavailability of the factor in the systemic circulation. Regardless of physiological state, the release kinetics of TGF-β showed a homogeneous profile: the concentration reached its maximum at T2, resulting in significantly higher levels compared to both the initial time point (T1) and the final time point (T4) (*p* < 0.05 for both comparisons). This confirms a rapid and standardized release of TGF-β in response to the stimulus.

For VEGF, a highly significant effect of Time was found (*p* < 0.0001), while the effects of Lactation Phase (*p* = 0.1173) and the interaction (*p* = 0.4497) were not significant. For IGF-1, no statistically significant effects were observed for Time (*p* = 0.6074), Lactation Phase (*p* = 0.2549) or the interaction (*p* = 0.1015). Given the non-significance of Phase and Interaction effects, the release kinetics are considered independent of the lactation phase. The peak release of VEGF was observed at T3, resulting in significantly higher levels compared to the T1 baseline (*p* < 0.05). The maximum IGF-1 concentration was measured at T3, resulting in significantly higher levels compared to T1 (*p* < 0.05).

## Discussion

To the best of the authors' knowledge, this study represents the first investigation into autologous l-PRF membranes derived from bovine blood. The study aimed to standardize a production protocol for l-PRF membrane in bovines, adapting the one previously standardized for dogs (Caterino et al., 2022), due to the absence of dedicated procedures for ruminants. Moreover, it aimed to explore the potential clinical usefulness of these membranes throughout a comprehensive analysis of their macroscopic and histological features, alongside the assessment of GFs release over time. The employment of regenerative medicine in bovine-intensive farming systems represents a real clinical challenge. Thanks to the documented healing and antimicrobic properties of the P-PRF (Aragosa et al. 2025; Kobayashi et al. 2016), the estimation of its effectiveness as an alternative method to manage some cows disease without resorting to antibiotics is an interesting future perspective. In this regard, the study fully addresses this need and adheres to the guidelines established by numerous international agencies, which unanimously indicate that the continuous and/or improper use of antibacterial molecules is an outdated practice and a genuine risk factor for the promotion of antibiotic resistance in livestock farms (FAO, 2021; ECDC, 2022). As reported by Guccione et al., (Guccione et al. 2024), to contribute to the “One Health” objective, the scientific community has, therefore, the ethical obligation to collaborate to be part of novel strategies for control prevention and treatment of clinical disease

Our investigation confirms the proposed centrifugation protocol's efficacy in producing l-PRF clots and membranes. Regarding the latter, the different stages, including a slow start, a fast phase in the middle, and a slow final phase proved to be effective and stable in generating membranes as reported in literature for other species (Caterino et al. 2022; Crisci et al. 2017; Dohan Ehrenfest et al. 2018). During the investigation, the authors were particularly careful in harvesting, storing, and centrifuging the samples within 2 min, as well as in assuring an inside centrifuge temperature in a range of 21–30 °C. As reported in literature, the temperature increases during the centrifugation stages over 30 °C and the blood sampling harvested over 2 min negatively impact the formation of l-PRF clots (Caterino et al. 2022; Crisci et al. 2017). Nevertheless, in our experience, the proposed protocol was easy to carry out, and the bovine blood sampling resulted feasible within the time laps considered. The study population was homogeneous; the sampling carried out during the springtime ruled out the seasonal effect of the platelet rate (Astuti, Widyobroto & Noviandi 2021).

The macroscopic and histological results confirm that a bovine venous blood sample quickly harvested and centrifuged produces a dense and well-organized l-PRF fibrin mesh. Indeed, the plasma and platelets are separated from the red cells and partially from the white one during the initial soft spin for 9 min at 2700 rpm (815 g), and 11 min at 2400 rpm (644 g). A second vigorous spin at 3000 rpm (1006 g) for 10 min condenses the fibrin and creates a rich fibrin structure distinguished by a dense fibrin clot. The sizes and weight of the bovine l-PRF membranes obtained by 15-minute compression of l-PRF clots in a Wound box commercially available make these suitable for clinical use. The histoarchitecture confirms the presence of five layers consisting of platelets, leukocytes, and red blood cells trapped in a fibrin mesh network. Indeed, during the synthesis of the l-PRF, the three-dimensional fibrin nano-scaffold allowed the incorporation of the platelets in a non-diffusible mode, binding platelet- and plasma-derived GFs before they attached to their corresponding cell-surface receptors. This typical l-PRF histoarchitecture is also described in the human, horse and canine models (Caterino et al. 2022; Crisci et al. 2017; Dohan et al. 2006b; Jiménez-Aristazábal, Carmona & Prades 2019). These results fulfil the evaluation required to standardize l-PRF production in bovine. The consistent release of GFs over time is well documented in humans as well as in dogs and cats (Caterino et al. 2022; Ghanaati et al. 2014); our results confirmed this trend in cattle as well.

The pivot role of l-PRF in tissue regeneration and wound healing lies in its anticipated properties, such as the high concentration of white blood cells, neutrophils and macrophages, which are among the first cells to appear at wound sites. These cells play critical roles in phagocytosing debris, microbes, and necrotic tissue, thereby preventing infections and potentially reducing the use of antimicrobials. Macrophages represent one of the key cells involved in growth factor secretion during wound healing, including transforming growth factor beta, platelet-derived growth factor, and vascular endothelial growth factor. These cells, in addition to neutrophils and platelets, are essential in wound healing and, thanks to their released growth factors and cytokines, may stimulate tissue regeneration, angiogenesis, and infection prevention. (Choukroun et al. 2006; Dohan et al. 2006c, 2006a, 2006b). Our LMM results demonstrated that the kinetic profiles of TGF-β1 and PDGF-AB differed significantly across lactation phases (significant Phase x Time), while the release kinetics of IGF-1 and VEGF were statistically independent of the lactation phase (non-significant interaction, *P* > 0.05). The TGF- β1, which plays a key role in immune response, tissue regeneration, and cell differentiation, in our sample showed a peak of expression at T2 (2 h) in all lactation phases, followed by a constant decrease over T3 (24 h) and T4 (72 h). This behaviour reflects its function stimulator of proliferation of various mesenchymal cell types, constituting the most powerful fibrosis agent among all cytokines involved in the early stages of the wound healing process.

In the late lactation group, otherwise, the marked reduction below baseline at T4 suggests rapid depletion or utilization. Probably due to the high rate of mammary gland reparative processes in which the PDGF-AA is involved (Lange-Consiglio et al. 2014).

An overlapping trend was detected, also, in the dry-off and early lactation. Indeed, the early significant release (T2 vs. T1) in the dry-off phase suggests a quick activation for involvement in the reparative and restoring process of the mammary gland (Lange-Consiglio et al. 2014). The PDGF-AB is a main regulator for the migration, proliferation, and survival of mesenchymal cell lineages. Indeed, its use in physiologic wound healing has been approved by the Food & Drugs Administration for the regeneration of various defects in medicine and dentistry (Border & Noble 1994; Howell et al. 1997). PDGF-AB is produced over time by leukocytes. It is considered one of the most important bioactive growth factors released over time and naturally present in l-PRF.

Due to the significant interaction (*p* = 0.0487), the release kinetic was phase-dependent (Table 3): its expression increased significantly up to T3 (24 h) in the early lactation group, reaching its maximum aggregated peak across all groups at T4 (48 h) in the Dry Off phase (5362.49 ng/mL), confirming the powerful role of l-PRF as a reservoir of this essential GF.

The TGF-β1, which plays a key role in immune response, tissue regeneration, cell differentiation, in our sample shows a homogeneous peak at T2 regardless of lactation phase, confirming a standardized rapid release mechanism for TGF-β1.

The TGF-β1 levels are significantly higher in the dry-off phase, suggesting that the period of mammary involution and rest is associated with a greater basal availability of this factor.

Since the TGF-β1 is a potent regulator of fibrosis, matrix formation, and immunomodulation, l-PRF from dry-off cows may be particularly effective in treating lesions where strong anti-inflammatory action is needed.

This behaviour reflects its function stimulator of proliferation of various mesenchymal cell types, constituting the most powerful fibrosis agent among all cytokines involved in the early stages of the wound healing process. In our study population, the VEGF and IGF-1 release showed a non-significant effect related to the lactation phase.

The IGF-1 is a positive regulator of proliferation and differentiation for most mesenchymal cells, also constitutes the major axis of programmed cell death (apoptosis) regulation (Giannobile et al., 1996). Since the release kinetic was independent of the lactation phase (EMMs aggregated), the overall profile showed a statistically significant increase with a peak at T3 (24 h) (2268.09 ng/mL), with a slight decrease at T4 (48 h).

Finally, VEGF is the most effective growth factor linked to tissue angiogenesis. (Shamloo et al., 2012). It has powerful effects on tissue remodelling, and its constant release aggregated across all phases, with a peak at T3 (24 h) (11.01 ng/mL) followed by a slight decrease overall time, could be related to its involvement in each phase of wound healing, independently of the lactation phase.

Our results showed GF behaviour in bovine l-PRF, supporting our hypothesis that l-PRF could enhance soft tissue wound healing, mainly through the enhancement of angiogenesis in defective areas, paving the way for new clinical studies applicable to regenerative medicine in this species, as has already been done for dogs, where a recent study has demonstrated its usefulness in the management of chronic wounds (Aragosa et al. 2025). Therefore, while confirming that the standardised protocol can produce l-PRF with sustained release of growth factors, our results represent relative rather than absolute release dynamics, because our evaluations were carried out on a bioscaffold and not on whole blood or clots.

At this point, it is important to emphasise that the proposed production protocol did not find any relevant negative influences on GF release due to different stages of lactation.

Moreover, the macroscopic and histological features of the l-PRF membranes confirm the effectiveness of the obtained 3D-bioscaffold for further clinical application. These findings support the proper standardization of the protocol, ensuring consistent results over time, and enabling its future clinical application regardless of the herd’s metabolic status. Nevertheless, l-PRF production presents some critical aspects that must be carefully managed to obtain a 3D-bioscaffold with optimal characteristics. Among these should be considered: (i) correct animal restraining during samplings, (ii) performing rapid blood collection, (iii) minimizing the time between sampling and centrifugation, (iv) maintaining appropriate centrifuge temperature, and (v) respecting the centrifugation protocol, particularly in terms of duration and speed. By the failure to respect these aspects, the fibrin architecture and the growth factor release may be negatively influenced, as recently observed in other species (Aragosa et al. 2025; Caterino et al. 2022). Further studies should be performed to refine these technical aspects, addressing the potential limitations and facilitating the clinical applications of l-PRF in bovine medicine.

## Conclusions

Considering the overall results regarding macroscopical, histological, and biological characteristics, the l-PRF membranes might be regarded as reliable for future use in bovine medicine to improve, enhance, and regulate the healing process. The encouraging results promote clinical trials in which all the characteristics observed might be tested and the clinical potential of bovine l-PRF membrane assessed. Additionally, exploring the antimicrobial properties of this autologous product could significantly advance its application in bovine healthcare, aligning with a One Health perspective.

## Glossary

**AB** = platelet-derived growth factor type AB

**BB** = platelet-derived growth factor type BB

**BP-*G*** = before peak-group

**DIM** = days in milk

***E*** = erythrocytes

**EL-*G*** = early lactation-group

***F*** = fibrin

**GFs** = growth factors

***L*** = leukocytes

**LL-*G*** = late lactation-group

**L-PRF** = Leukocyte-Platelet-Rich Fibrin

**ML-*G*** = middle lactation-group

***P*** = platelets

**PCs** = Platelet Concentrates

**PDGF-AA** = platelet-derived growth factor type AA

**PPP** = platelet-poor plasma

**PRF** = Platelet-Rich Fibrin

**PRP** = Platelet-Rich Plasma

**P-PRF** = Pure Platelet-Rich Fibrin

**P-PRP** = Pure Platelet-Rich Plasma

**RBC** = red blood cells

**SD** = standard deviation

**SEM** = standard error of the mean

**TGF-β1** = transforming growth factor-β1

**VEGF** = vascular endothelial growth factor

## Data availability

The data that support the findings of this study are available from the corresponding author upon reasonable request.

## Data integrity statement

All authors have full access to all data in the study and take responsibility for the integrity of the data and accuracy of data analysis.

## Ethical animal research

The sampling received institutional approval from the Ethical Animal Care and Use Committee of the university of Napoli Federico II (pg/2017/0099607).

## Funding sources

This research did not receive any specific grant from funding agencies in the public, commercial, or not-for-profit sectors.

## Informed consent

Informed consent was obtained from the farmer involved in the study.

## CRediT authorship contribution statement

**Giovanni Della Valle:** Writing – original draft, Validation, Supervision, Methodology, Investigation, Formal analysis, Data curation, Conceptualization. **Maria Chiara Alterisio:** Writing – review & editing, Writing – original draft, Visualization, Validation, Methodology, Investigation, Formal analysis, Data curation, Conceptualization. **Jacopo Guccione:** Writing – review & editing, Writing – original draft, Visualization, Validation, Supervision, Methodology, Investigation, Formal analysis, Data curation, Conceptualization. **Chiara Caterino:** Writing – review & editing, Writing – original draft, Visualization, Investigation, Data curation, Conceptualization. **Federica Aragosa:** Writing – original draft, Visualization, Formal analysis, Data curation, Conceptualization. **Gianmarco Ferrara:** Writing – review & editing, Validation, Software, Formal analysis, Data curation. **Davide De Biase:** Writing – review & editing, Validation, Formal analysis, Data curation. **Paolo Ciaramella:** Writing – review & editing, Visualization, Validation, Supervision, Methodology. **Gerardo Fatone:** Writing – review & editing, Visualization, Validation, Supervision, Methodology, Investigation, Conceptualization.

## Declaration of competing interest

The authors declare that they have no known competing financial interests or personal relationships that could have appeared to influence the work reported in this paper.

## Appendix Supplementary materials

**Supplementary File 1** Title: Criteria employed to exclude any diseases in the enrolled animals during the entire study period from a previous and partially modified study. Image, application 1
