# Supplementary material for: New frontiers in regenerative medicine: Protocol standardization and morphological assessment of leukocyte-platelet rich fibrin in cows, with a longitudinal study on growth factor release dynamics
Source: Vet Anim Sci. 2025 Dec 29;31:100564. doi: 10.1016/j.vas.2025.100564 (PMC12807813; doi:10.1016/j.vas.2025.100564)
Supplement: Supplementary file 1 — Title: Criteria employed to exclude any diseases in the enrolled animals during the entire study period from a previous and partially modified study. [file mmc1.docx]

### Supplementary file 1

**Leukocytes-platelets rich fibrin preparation method: protocol standardization, macroscopic and histologic evaluations, and growth factors assessment in dairy cows.**

G. Della Valle ^1^, M. C. Alterisio ^1^, J. Guccione ^1^*, C. Caterino ^1^, F. Aragosa ^1^ G. Ferrara ^2^, D. De Biase ^3^, P, Ciaramella ^1^, G. Fatone ^1^

^1^ Department of Veterinary Medicine and Animal Production, University of Naples “Federico II”, Naples, Italy

^2^ Department of Veterinary Sciences, University of Messina, Italy

^3^ Department of Pharmacy/DIFARMA, University of Salerno, Fisciano, Italy

Criteria employed to exclude any diseases in the enrolled animals during the entire study period from a previous and partially modified study (Fadul et al., 2022).

| **Pathologies** | **Clinical**-**diagnostic procedures** |
| --- | --- |
| **Claw and foot disorders-diseases^a,b,c,e^** | - Locomotion score (lame if LS< 2.5). |
| **Ketosis^a,c,e,g,h,i^** | - General clinical examination (to assess overall health status). - Body condition score (to assess if the body condition was within the targets). - Blood beta-hydroxybutyrate > 1.4 mmol/l in lactating cows (4-5 h after the start of feeding). - Blood glucose < 3.0 mmol/l for transition cows (during the entire study-period, 4-5 h after the start of feeding). |
| **Subacute ruminal acidosis^a,c,e,g^** | - General and specific clinical examinations (to assess overall and gastro-intestinal health status) - Faecal consistency (score below 3) and faecal sieve test (particles > 0.5 cm). |
| **Subclinical Hypocalcemia^a,c,e,f,g,^** | - General and specific clinical examinations (to assess overall and gastro-intestinal health status) - Body condition score (to assess if the body condition was within the targets) - Absence of recumbency - Blood total blood iCa^2+^ ≤1.0 mM (during the entire study-period) |
| **Cecum dilation^a,c,e^** | - General and specific clinical examinations (to assess overall and gastro-intestinal health status) - Blood gas analysis (to assess the acid-base status) |
| **Abomasum displacement^a,c,e^** | - General and specific clinical examinations (to assess overall and gastro-intestinal health status) - Blood gas analysis (to assess the acid-base and electrolyte status) |
| **Tracheobronchitis^a,c,e^** | - General and specific clinical examinations (to assess overall and respiratory system health status) - Blood gas analysis (to assess pulmonary gas exchange and acid-base status status) - Complete blood cell count |
| **Puerperal Metritis^a,c,d,e^** | - General clinical examination and trans-rectal palpation (to assess overall and female genital system status) - Timing of the problem (≤21 days of calving) - Vaginal examination and uterine discharge observation (fetid and red brown) - Complete blood cell count |

LS=locomotion score; iCa=ionized calcium; ^a^Dirksen (2004); ^b^Egger-Danner et al., (2016); ^c^Fubini and Divers (2008); ^d^Hudson (2012);^e^Jackson and Cockcroft (2002); ^f^Martinez et al., (2014); ^g^Mulligan et al., (2006); ^h^Oetzel (2004), ^i^Whitaker (1997)

**References**

Dirksen, G. 2004. Malattie del reticolo e del rumine nel bovino ruminante. Pages 396-454 In Medicina Interna e Chirurgia del Bovino. Dirksen G., Grunder H.D., Stober M. Ed. Le Point Veterinaire Italie srl, Milano, IT.

Egger-Danner C, Nielsen P, Fiedler A, Müller K, Fjeldaas T, Döpfer D, et al. ICAR Claw Health Atlas. 2015. http://www.icar.org/wp-content/uploads/ 2016/02/ICAR-Claw-Health-Atlas.

Fadul M, D'Andrea L, Alsaaod M, Borriello G, Di Lori A, Stucki D, Ciaramella P, Steiner A, Guccione J. 2022. Assessment of feeding, ruminating and locomotion behaviors in dairy cows around calving - a retrospective clinical study to early detect spontaneous disease appearance. PLoS One. 2022 Mar 4;17(3):e0264834.

Fubini, S. and T. J. Divers. 2008. Non-infectious Diseases of the Gastrointestinal Tract. Pages 130-199 In Rebhun's - Disease of Dairy Cattle. Divers T.J. and Peek S.F. Ed. Saunders Elsevier Westline Industrial Drive St. Louis, Missouri, USA.

Hudson, C., M. Kerby, J. Statham and W. Wapenaar. 2012. Managing Herd Reproduction. Pages 108-110 In Dairy Herd Health. Green M., Bradley A., Breen J., Higgins H., Hudson C., Huxley J., Statham J., Green L., Hayton A. Ed. Martin Green, University of Nottingham, UK.

Jackson, P. and Cockcroft, P. 2002. The General Clinical Examination of Cattle. Pages: 9-11. In Clinical Examination of Farm Animals. 1st ed. John Wiley & Sons, New York, NY.

Mulligan, F.J, O'Grady, L., Rice, D.A., and M.L. Doherty. 2006. A herd health approach to dairy cow nutrition and production diseases of the transition cow. Anim Reprod Sci. 2006 96:331-53.

Martinez N, Sinedino LD, Bisinotto RS, Ribeiro ES, Gomes GC, Lima FS, Greco LF, Risco CA, Galvão KN, Taylor-Rodriguez D, Driver JP, Thatcher WW, Santos JE. 2014. Effect of induced subclinical hypocalcemia on physiological responses and neutrophil function in dairy cows. J Dairy Sci. 97:874-87.

Oetzel, G.R., 2004. Monitoring and testing dairy herds for metabolic disease. Vet. Clin. Food Anim. Pract. 20, 651–674.

Whitaker, D.A., 1997. Interpretation of metabolic profiles in dairy cows. Cattle Pract. 5 (1), 57–60.
